# A Disintegrin and Metalloprotease with Thrombospondin Motif, Member 13, and Von Willebrand Factor in Relation to the Duality of Preeclampsia and HIV Infection

**DOI:** 10.3390/ijms26094103

**Published:** 2025-04-25

**Authors:** Prelene Naidoo, Thajasvarie Naicker

**Affiliations:** Optics & Imaging Centre, Doris Duke Medical Research Institute, University of KwaZulu-Natal, 719 Umbilo Road, Congella, Durban 4013, South Africa

**Keywords:** HIV, preeclampsia, ADAMTS13, VWF, pregnancy

## Abstract

Normal pregnancy is associated with multiple changes in the coagulation and the fibrinolytic system. In contrast to a non-pregnant state, pregnancy is a hypercoagulable state where the level of VWF increases by 200–375%, affecting coagulation activity. Moreover, in this hypercoagulable state of pregnancy, preeclampsia is exacerbated. ADAMTS13 cleaves the bond between Tyr1605 and Met1606 in the A2 domain of VWF, thereby reducing its molecular weight. A deficiency of ADAMTS13 originates from mutations in gene or autoantibodies formed against the protease, leading to defective enzyme production. Von Willebrand protein is critical for hemostasis and thrombosis, promoting thrombus formation by mediating the adhesion of platelets and aggregation at high shear stress conditions within the vessel wall. Mutations in VWF disrupts multimer assembly, secretion and/or catabolism, thereby influencing bleeding. VWF is the primary regulator of plasma ADAMTS13 levels since even minute amounts of active ADAMTS13 protease have a significant inhibitory effect on inflammation and thrombosis. VWF is released as a result of endothelial activation brought on by HIV infection. The SARS-CoV-2 infection promotes circulating proinflammatory cytokines, increasing endothelial secretion of ultra large VWF that causes an imbalance in VWF/ADAMTS13. Raised VWF levels corresponds with greater platelet adhesiveness, promoting a thrombotic tendency in stenotic vessels, leading to increased shear stress conditions.

## 1. Introduction

Maternal mortality is the annual number of maternal deaths emanating from any cause connected to or aggravated by pregnancy or its management during pregnancy and childbirth or within 42 days of the termination of pregnancy [1]. Of note, 95% of all maternal deaths predominated in low- and middle-income countries [1]. In the period 2000–2020, the maternal mortality ratio (MMR) (number of maternal deaths per 100,000 live births) decreased across the world by 34% [2].

In 2020–2021, the MMR in South Africa (SA) was 120.9 maternal deaths per 100,000 live births. There has been a slight decrease in MMR (119.1 deaths per 100,000 live births) during the period 2021/2022 in SA. However, the MMR currently in SA is 125 deaths per 100,000 live births [3,4]. The leading cause of maternal deaths in SA is non-pregnancy-related infections such as HIV, tuberculosis, pneumonia, etc., followed by hemorrhage, medical, and surgical disorders [4]. The fourth and direct leading cause of maternal deaths in SA is hypertensive disorders of pregnancy (HDP) of which 83% is due to preeclampsia (PE) and eclampsia [5].

Hypertensive disorders of pregnancy affects 4–8% of all pregnancies worldwide [6]. Globally, it is also responsible for over 500,000 fetal and neonatal deaths and over 70,000 maternal deaths [7]. In SA, the prevalence of PE is 12% and is associated with 15% of preterm births [8]. Notably, a previous history of PE affects one’s life expectancy due to an increased risk of cardiovascular disease, diabetes, and the development of stroke later in life [9].

Preeclampsia is a pregnancy specific condition characterized by new onset hypertension at ≥20 weeks of gestation and is associated with either/or proteinuria; maternal end-organ dysfunction, including neurological complications; pulmonary edema; placental abruption; angiogenic imbalance; fetal growth restriction; and intrauterine fetal death [7]. It has a variable clinical presentation associated with decreased uteroplacental flow emanating from defective placentation [10] and coagulation abnormalities that may/may not result in a myriad of clinical abnormalities of the fetus [11,12]. Definitive treatment for PE is the delivery of the placenta [13] and effective treatment is premature delivery or termination of the pregnancy [14].

Pregnancy is often referred to as a hypercoagulable state characterized by increased levels of coagulation factors, decreased fibrinolysis, and increased platelet activation [15]. These changes are primarily due to the production of procoagulant molecules by the placenta, which serve to protect the mother from excessive blood loss during childbirth [16]. The hormonal changes in pregnancy also contribute to this hypercoagulable state [17]. Normal pregnancy is associated with the dysregulation of coagulation [15] and fibrinolytic activity across trimesters [18]. The levels of Von Willebrand Factor (VWF) may increase by 200–375% in pregnancy, often resulting in the doubling of coagulation compared to a non-pregnant state [15]. Notably, platelet count often decreases because of its consumption by the uteroplacental unit [15]. The dysregulation of coagulation in pregnancy may lead to various complications such as disseminated intravascular coagulation, a condition characterized by excessive clotting throughout the body, leading to organ failure [19]. As a typical physiological reaction, VWF rises and a disintegrin and metalloprotease with thrombospondin type 1 motif 13 (ADAMTS13) falls during pregnancy [20]. Abnormal persistence of ultra-large VWF (ULVWF) multimers is caused by severe ADAMTS13 deficiency, which triggers thrombotic thrombocytopenic purpura (TTP), thereby predisposing microvascular thrombosis [21].

In PE, the hypercoagulable state is accentuated [18]. The intrinsic coagulation pathway is activated and the common pathway appears to be hypercoagulable in PE [18]. More specifically, prothrombin times are accelerated in PE, linked to alterations in fibrinogen and factors II, V, and XI [22]. In PE, tissue type plasminogen activator, synthesized by endothelial cells, are released during endothelial injury thereby triggering the fibrinolytic system [23,24]. Hematological abnormalities are indicative of intravascular coagulation and less frequently erythrocyte destruction in PE [18].

There is a complex interplay between HIV infection and coagulation processes. HIV not only affects the immune system but also induces a proinflammatory state that can lead to hypercoagulability [25]. Patients with HIV are at a higher risk for thrombo-embolic events, which are exacerbated by factors such as chronic inflammation and antiretroviral therapy (ART) side effects [26]. Antiretroviral therapies, whilst essential for managing HIV infection, can also influence coagulation profiles, thus complicating patient care [27]. Additionally, emerging evidence suggests that the presence of HIV can alter platelet function and increase levels of clotting factors, further complicating the coagulation cascade [27].

## 2. A Disintegrin and Metalloprotease with a Thrombospondin Motif Type 1 Member 13 (ADAMTS13)

All mammalian genomes contain nineteen genes of A Disintegrin And Metalloprotease ThromboSpondin type motif (ADAMTS) [28]. This gene belongs to the metzincin protease superfamily, and it lacks both epidermal growth factor-like transmembrane and cytoplasmic modules [28]. ADAMTS13 has a number of unique properties for a circulating protease: 1. ADAMTS13 is constitutively secreted as an active protease [29]. 2. Relative to other coagulation and ADAMTS proteases, ADAMTS13 has a prolonged circulating half-life (two to four days), due in part to a lack of physiological inhibitors [30]. 3. The only known substrate of ADAMTS13 is VWF, and this protease displays no known off-target proteolysis [29].

Localization: ADAMTS13 is localized on chromosome 9q34 which contains twenty-nine exons spanning 37 kb in the genomic sequence [31,32]. It is synthesized primarily in the liver [32]. Its messenger RNA and translated proteins are localized to hepatic stellate cells [33]. In human plasma, the concentration of ADAMTS13 is 0.7–1.4 μg/mL [34].

Function: ADAMTS13 is known to process the large multimeric VWF precursor protein under fluid shear stress conditions [28]. The VWF-A2 domain and these domains have a complex exosite interaction that controls substrate specificity and cleavage efficiency [31,33,35]. The cleavage of a single peptide bond (Tyr1605-Met1606) on the A2 domain of the VWF molecule generates VWF proteins required for the maintenance of hemostasis [31].

Structure: Structure–function analysis has established that the ADAMTS13 fragment consists of the signal peptide (Sp), a propeptide (P; pro-domain), metalloprotease domain (M), disintegrin domain (D), first thrombospondin 1 repeat domain (Tsp1), Cys-rich domain (C), and spacer domain (S) at the N-terminal with seven additional Tsp1 repeat domains and 2 CUB domains (Complement c1r/c1s, sea Urchin epidermal growth factor, and Bone morphogenetic 1 and 2 proteins) [31,32] (Figure 1). Mutations in the highly variable regions of these domains significantly impair ADAMTS13 function [36,37].

Notably, the Sp component serves to guide the enzyme to the endoplasmic reticulum, where it undergoes post-translational modification and folding before being transported to the Golgi apparatus for further processing and eventual secretion outside the cell [38,39]. During this process, the Sp is cleaved off, thereby releasing the mature form of ADAMTS13 into the extracellular space where it can perform its enzymatic function [38]. The P component is involved in maintaining the enzyme in an inactive state while inside the cell and upon secretion into the bloodstream, proteolytic cleavage removes this P component, thereby activating ADAMTS13 and allowing it to cleave VWF efficiently [40].

The M domain on ADAMTS13 alone does not have the ability to bind with specificity to VWF or to cleave at the VWF cleavage site [29]. This non-catalytic domain is necessary for substrate specificity [41]. ADAMTS13 activity includes the binding of three calcium ions which is essential for the structural integrity of the proteolytic site [42]. Moreover, it has several hydrophobic residues that ensure the protease binds to VWF [34]. This M domain contains no crystal structures but is characterized by the adamalysin/reprolysin type, zinc-binding sequence (HEXXHXXGXXHD), where H denotes histidine, E glutamic acid, G glycine, D aspartic acid, and X variable amino acid [38,43]. Zinc cations must be bound for enzymatic activity since zinc-coordinating and calcium-binding residues control the cleavage activity of ADAMTS13 [44]. Also, leu1603 is a residue present on the VWF molecule that interacts with residues near the zinc ion allowing for proper cleavage of VWF [32].

The next domain on ADAMTS13 is the D domain which lacks the cysteine signature or the arginine, glycine, asparagine motif but contains a crystal structure. The M and D domains are functionally coupled [28,32]. The addition of this short D domain restores full substrate binding specificity and specific proteolytic activity to the cleavage site of the M domain [34]. Within this domain, two hydrophobic residues and one charged residue have been identified and binds to VWF [34].

The first Tsp1 repeat domain on ADAMTS13 has three anti-parallel strands; the whole domain is capped by disulfide bonds on each end [32]. These strands are stabilized by a cysteine-, tryptophan-, and arginine-layered core [45]. The Tsp1 domain engages in substrate recognition and functions in facilitating ligand interaction [31,46].

The C domain is structurally similar to the D-domain. This domain has a short α-helix and two pairs of double stranded anti-parallel β-sheets stabilized by six disulfide linkages [32]. De Groot hypothesized that this domain may contribute specifically to ADAMTS13 function [47]; however, other studies have only implicated its role in the interaction with VWF [48,49]. Nonetheless, this domain is homologous with others in the ADAMTS family; however, the non-conserved V-loop forms a hydrophobic pocket to favor interactions with the VWF A2 domain [50]. The residues Gly471-Val474 at the base of the variable loop within the cysteine-rich domain forms a hydrophobic pocket involved in the binding to hydrophobic residues on VWF [34,47]. The absence of these hydrophobic interactions result in a 75-to-200-fold decrease in proteolysis [47].

The S domain differs across members of the ADAMTS family and is critical for substrate recognition; this domain has the highest binding affinity for the A2 site in VWF [12,32,34]. Residues Leu621-Asp632 in the spacer domain form a loop that interacts with the proximal portion of the cysteine-rich domain [48]. Both the C and S domains have been suggested to function closely and similarly to each other [34]. The S domain acts as a connector between the functional domains, potentially influencing enzyme activity, while the M domain plays a direct role in substrate recognition and binding [34,48].

The seven additional Tsp1 repeats at the C-terminal have no available crystal structure; they are required largely for flexibility and supporting the two CUB domains and S- domain interaction which maintains latency [50]. Of note, the deletion of Tsp1 (7) and Tsp1 (8) disrupts allosteric regulation [34].

The two CUB domains at the C-terminus are unique to ADAMTS13. The crystal structure of the CUB domains have approximately 110 residues in anti-parallel stranded β-sheets, characteristic of a β-sandwich structure [51]. Furthermore, ADAMTS13 CUB domains lack calcium-binding and N-linked glycan [51,52]. Linker regions exist between Tsp1 (2–5)- and Tsp1 (8)-CUB domains, which provide the necessary flexibility to the Tsp1 repeats in the tail allowing ADAMTS13 to adopt the closed conformation [53]. The two CUB domains are involved in the initial binding between ADAMTS13 and the D4-CK domain of VWF [54]. This results in conformational changes in both proteins, resulting in the linearizing of VWF and unfolding of ADAMTS13 through the disruption of the spacer–CUB interaction [50]. Mutations in both CUB domains affects protein secretion rather than directly affecting protease activity [50]. The two CUB domains are also involved in developmental regulation [51].

## 3. Von Willebrand Factor

Von Willebrand Factor is a large multimeric glycoprotein that controls platelet adhesion and aggregation [35] with an average plasma concentration of ~10 μg/mL [32,34]. It is interesting to note that VWF multimers can be cleaved by plasmin, another protease found in plasma [55]. In the area of the polypeptide chain connecting domains A1 and A2, the connection between amino acid residues K1491-R1492 is broken by plasmin [56]. The equivalent of vasopressin, desmopressin, causes an immediate 2-fold rise in the amount of VWF antigen in human blood plasma by substituting D-arginine for L-arginine via V2 receptors [55].

Localization and storage: Following extensive post-translational processing such as glycosylation, sulfation, and assembly, each mature VWF monomer contains 2050 amino acids made up of conserved domains [32]. It is produced primarily in vascular endothelial cells and α-granules of platelets and is stored in either the Golgi apparatus of endothelial cells or within endothelial cytoplasmic granules referred to as Weibel–Palade bodies (WPBs) and is also found within subendothelial connective tissue [57]. Following synthesis, VWF is transported to storage organelles in both megakaryocytes/platelets (α-granules) and endothelial cells (Figure 2) [58].

The majority of VWF is secreted constitutively, whereas the remainder is stored in WPBs that are specific for endothelium [60]. The WPBs and α-granules differ from each other in their dependency on VWF for their formation: α-granules can form in the absence of VWF, whereas the generation of WPBs is strictly VWF dependent [61]. Circulating VWF in plasma is predominantly endothelial cell derived, as platelets release VWF from α-granules only when activated [62]. There are some key differences between VWF originating from endothelial cells versus megakaryocytes. Endothelial cell-VWF is constitutively secreted and undergoes proteolytic processing by ADAMTS13, whereas platelet-VWF is not constitutively secreted and does not undergo significant proteolysis [63].

Function: Under shear stress conditions, the VWF protein promotes thrombus formation by mediating the adhesion of platelets and aggregation within vessels [35]. Additionally, it carries and stabilizes coagulation factor VIII (FVIII) in circulation and acts as the main adhesive link between platelets and the subendothelium [31]. Numerous plasma and matrix proteins can interact with the VWF subunit, such as the collagen matrix and coagulation factor VIII, which interact with the A1 domain of VWF and platelet glycoprotein 1b with the A3 domain of VWF, respectively [32]. Also, VWF carries a procoagulant FVIII, which protects it from rapid proteolytic degradation and delivery to sites of vascular damage [64]. Notably, VWF is released into the bloodstream, serves as a carrier protein for blood clotting factor VIII, and enables platelets to adhere to the damaged vascular wall by interacting with collagen [61,65].

The multimeric size of VWF is central to its platelet-tethering function, with larger multimers conferring greater hemostatic potential than smaller forms [47]. The length and thickness of VWF multimers strongly correlate with physiological hemostatic potential, making its cleavage by ADAMTS13 critical for balanced hemostasis [32,33]. Secretion of VWF multimers are induced by thrombin, histamine, fibrin, complement protein C5b-9 complexes, and bacterial *Shiga* toxin at the site of vascular injury [33].

Structure: The VWF sequence contains an unusually high content of cysteine residues paired by disulfide bonds in all domains except in the A domains [32,34,66]. Cysteine plays a critical role in stabilizing domain structure [67]. The mature subunit is extensively glycosylated with 12 N-linked and 10 O-linked oligosaccharides, whilst the propeptide has three more potential N-glycosylation sites [32,34].

The domains of VWF are arranged in the following sequence: D1-D2-D’-D3-A1-A2-A3-D4-B1-B2-B3-C1-C2-CK [52]. Domains D1-D2-D’-D3-A1-A2-A3-D4-C1-C2-C3-C4-C5-C6-CK create the first VWF monomer [55]. The following structural domains are present in the mature VWF subunit: D’-D3-A1-A2-A3-D4-C1-C2-C3-C4-C5-C6-CK [68] (Figure 3). The following modules are part of Domains D1, D2, and D3: Trypsin inhibitor-like (TIL), cysteine-8 (C8), von Willebrand D domain (VWd), and E modules (E) [52]. The VWd domain and C8 module are absent from Domain D’, although D4 contains a D4N subdomain and no E-module [55]. The C-terminal cysteine knot (CK) domain dimerizes in the endoplasmic reticulum, while the D1, D2, D’, and D3 domains regulate the assembly of di-sulfide linkage of VWF dimers in the acidic environment of the Golgi apparatus [69].

Triplicated A domains form the VWF monomer’s essential constituent. Each of the A1 and A3 domains is fixed in a rather stiff structure by a disulfide bridge formed by its N- and C-terminals. In contrast, the A2 domain is flexible and susceptible to proteolysis because it stretches in high shear stress situations due to its non-rigid structure [55,70]. Of note, VWF ultra-large multimers are more thrombogenic [55].

The primary function of the A1 domain of VWF is to capture platelets via GPIbα, resulting in the formation of the platelet plug [55,71]. In addition to platelet binding, the A1 domain can also bind heparin, and this can competitively inhibit platelets from binding to VWF, thereby affecting platelet plug formation during heparin administration [72]. The A1 domain has also been shown to interact with collagen IV in the basement membrane [73]. The A2 domain contains a calcium-binding loop in the α3-β4 loop, which protects it against cleavage by ADAMTS13 by promoting rapid refolding of the domain [70]. The Tyr1605-Met1606 cleavage site for ADAMTS13 is buried in the hydrophobic core of the β4-sheet [70]. The β4-sheet, which contains the ADAMTS13 cleavage site, is where the A2 domain unfolds under shear tension, starting at the C-terminus [74]. When vascular injury occurs, the A3 domain makes it easier for VWF to attach to the exposed subendothelial collagen [72]. Moreover, the A3 domain also interacts with collagen sequences containing positively charged and hydrophobic residues [75].

The C-terminal domains of VWF play an important role in binding to ADAMTS13. More specifically, D4, C1–C6, and CK domains of VWF interact with the C-terminal domains of ADAMTS13 [Tsp1 (5–8) and CUB domains (CUB1 and CUB2)] [67]. These C-terminal domains of VWF allow ADAMTS13 to bind and form a complex with VWF and circulate in plasma [67]. The formation of this complex is a critical step in the proteolysis of VWF by ADAMTS13 in circulation [67]. The CK domain plays an important role in the dimerization of VWF, which is required for the formation of long multimers [76]. In each monomer, the CK domains flank the inter-chain disulfide bonds and the backbone of hydrogen bonds of the β-sheet, creating a rigid cross-linked structure that is highly resistant to hydrodynamic forces, which may contribute to the efficient transmission of force between monomeric subunits in the VWF multimer [76].

## 4. Interactions Between Adamts13 and Von Willebrand Factor

**Figure 4:**
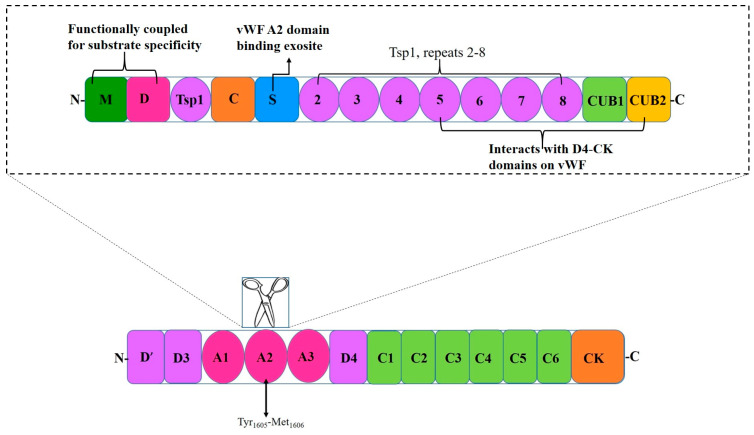
Illustration of ADAMTS13 cleaving VWF in the A2 domain. M—metalloprotease domain; D—disintegrin domain; C—cysteine rich domain; S—spacer domain; Tsp—thrombospondin motif domain (1–8).

**Figure 5:**
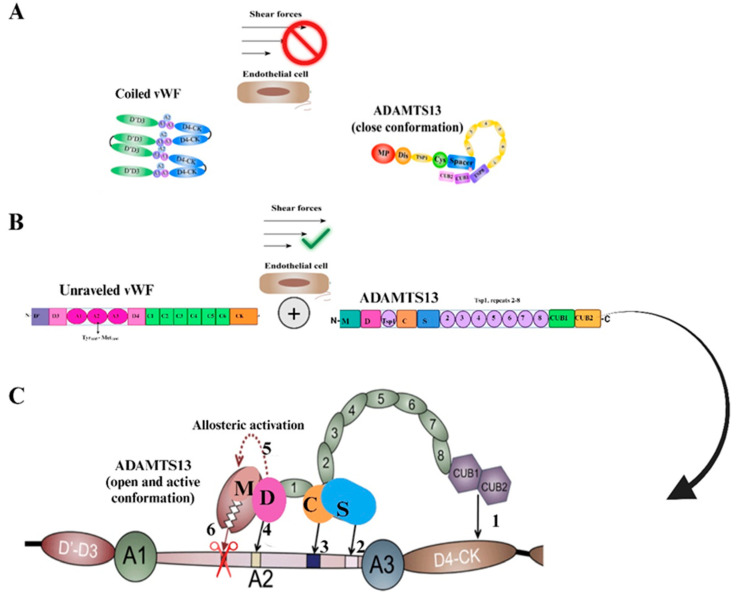
Schematic diagram showing the interactions between ADAMTS13 and VWF: (**A**) Under typical conditions, multimeric VWF moves around in the plasma in a globular shape. The interaction of the CUB domains with the spacer domain stabilizes the “closed” conformation in which ADAMTS13 circulates; shear forces are absent. (**B**) The binding site (A2) and cleavage site (Tyr_1605_-Met_1606_) for ADAMTS13 are revealed by unraveling VWF in the presence of shear stresses. (**C**) ADAMTS13 uses several interactions in the sequence from 1 to 6 to identify unfolded VWF. The domains of ADAMTS13 are M—metalloprotease, D—disintegrin, C—cysteine rich, S—spacer, and Tsp—thrombospondin motif (1–8); adapted from [45,81].

**Figure 1:**
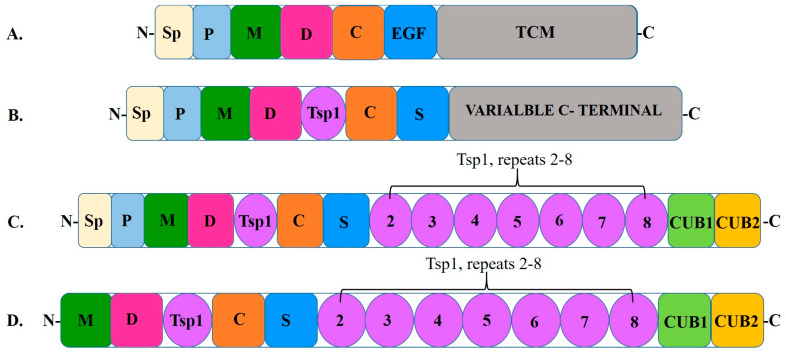
Schematic diagram of (**A**)—ADAM, (**B**)—ADAMTS, and (**C**)—ADAMTS13 with propeptide attached and (**D**)—a Mature ADAMTS13 structure. The structural domains are indicated as follows: signal peptide (Sp), propeptide (P), metalloprotease (M), disintegrin domain (D), first thrombospondin type 1 (Tsp1), cysteine-rich domain (C), spacer domain the second to eighth Tsp1 repeats 2–8, and two CUB domains (CUB1 and CUB2).

**Figure 2:**
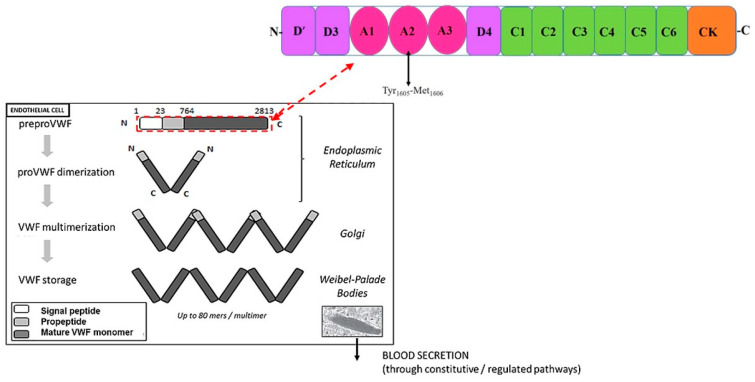
Schematic illustration representing VWF storage and structure. The prepro-VWF undergoes dimerization followed by multimerization and is finally stored in Weibel–Palade bodies (adapted [59]).

**Figure 3:**
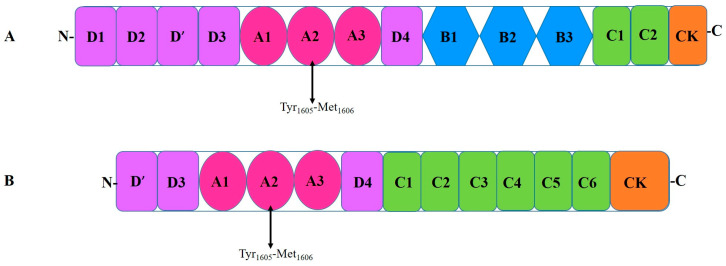
Von Willebrand factor structural arrangement: (**A**)—VWF with propeptide, (**B**)—mature VWF structure. The Tyr-Met bond in the A2 domain is cleaved by ADAMTS13. Domains of VWF are arranged in the following sequence: D1-D2-D’-D3-A1-A2-A3-D4-B1-B2-B3-C1-C2-CK.

When VWF is unwinding, the exosite that binds to the S domain is initially exposed, and this allows the S domain to recognize the VWF exosite [31,32]. ADAMTS13 holds a closed conformation maintained by the interaction between the S domain and C-terminal domains. Once the VWF D4-CK domains bind to the CUB1 and CUB2 domains of ADAMTS13, a conformational change occurs on ADAMTS13, transforming it from a closed to an open state. This exposes the S domain exosite therefore facilitating binding to the unfolded VWF A2 domain [77]. The S domain and the C domain function closely with and similarly to one another. When the Tsp1 (2–8) repeats and the CUB domains are truncated, the remaining domains still cleave VWF substrates [32,35]. The CUB domains alone have no measurable affinity for VWF; however, in the presence of shear stress, the CUB1 peptide will inhibit proteolysis of VWF [32]. Five thiol groups within Tsp1 repeats 2–8 and CUB-1 domain form disulfide bonds with VWF, therefore anchoring the enzyme; these free thiol interactions of the distal regions have anti-thrombotic activity independent of the proteolytic functions of ADAMTS13 [34]. The CUB domains have a negative regulatory function of ADAMTS13 activity; however, they may also have regulatory functions entirely unrelated to the proteolytic activity [32] (Figure 4 and Figure 5).

Also, platelet VWF, which is present in a high molecular weight state, is resistant to ADAMTS13 proteolysis because it does not have N-linked sialylation [78]. VWF is less vulnerable to cleavage by ADAMTS13 when calcium binds to the A2 domain, preventing unfolding by denaturants and encouraging refolding under a tensile strain [79]. Having a dissociation constant (KD) of about 20 nM, ADAMTS13 binds soluble VWF adsorbed onto the cell surface, thereby cleaving ULVWF bundles [32]. The cleavage of VWF can occur in the absence of flow but is slightly enhanced by fluid shear stress, therefore cell bound ULVWF is in its “open” conformation [33,34]. Until arterial shear is applied, re-released ULVWF in solution displays a “closed” conformation that is resistant to ADAMTS13 cleavage [28,31,32,33,80]. The rate of soluble VWF’s proteolysis by ADAMTS13 is dramatically increased when platelet glycoprotein 1b and/or FVIII bind to it under shear stress conditions [33,45]. By altering domain–domain interactions and destabilizing the cleavage site located in the VWF-A2 domain, GP1b and FVIII exert a rate-enhancing effect on VWF proteolysis [32]. Endothelium-anchored ULVWF strings can collect platelets in the absence of ADAMTS13 activity, which can result in uncontrollable thrombosis in capillaries and terminal arterioles [32]. The closed conformation is relieved when distal C-terminal domains of ADAMTS13 interact with the distal domains of VWF (D4-CK), resulting in exposure of the spacer domain, which in turn engages the A2 domain of VWF (Figure 4 and Figure 5) [54].

## 5. Preeclampsia

The highest prevalence of HDPs predominates in South Asia (3.84 million), western sub-Saharan Africa (3.71 million), and eastern sub-Saharan Africa (3.12 million) [82]. These women from low-income countries such as South Asia and sub-Saharan Africa [83] are at a higher risk of developing PE compared to those in high-income countries [84]. Of note, sub-Saharan Africa (56%) and South Asia account for 85% of the global burden of deaths [82].

Based on the onset of clinical signs and symptoms, PE may be categorized into two sub-types viz., early-onset PE (EOPE) occurring <34 weeks of gestation and late-onset PE (LOPE) that occurs >34 weeks of gestation [9,85]. The EOPE is characterized by defective placentation and is associated with severe clinical manifestations to both mother and baby [85,86]. In contrast, LOPE is referred to as a maternal disorder characterized with endothelial injury [85]. Of note, intrauterine growth restriction dominates in EOPE [87]. Severe PE predisposes to intravascular coagulation, increasing the risk of bleeding [18].

Preeclampsia has a multifactorial pathogenesis where the placenta is the main organ affected [11]. Its pathophysiology is linked to vascular, immunologic, and genetic factors that culminate in multiple-organ injury, including the liver and kidney [88]. Placental villous lesions are found in 45.2% and 14.6% of PE and normotensive pregnancies, respectively [24]. Maternal age above 35, obesity, a history of PE [89], racial background, an elevated body mass index (BMI) [90], primiparity, and underlying medical disorders such diabetes mellitus, chronic hypertension, and heart and kidney diseases are all risk factors for PE development [91].

**Figure 6:**
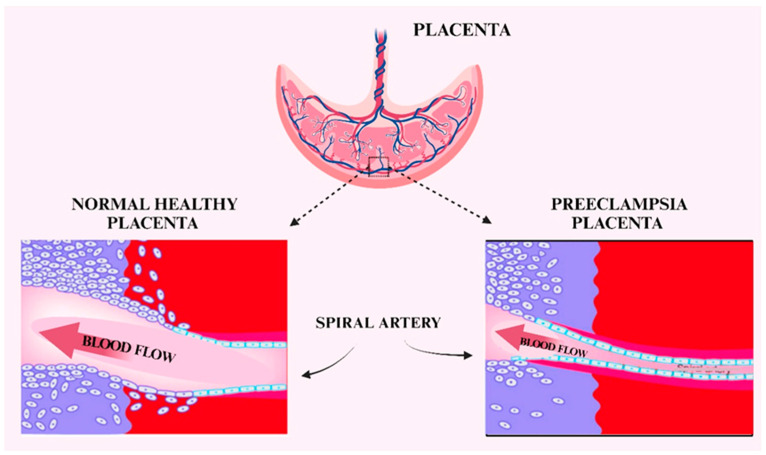
The spiral artery in normal pregnancies vs. the spiral artery in preeclampsia. In order to guarantee that the uterine blood vessels carry enough blood for the fetus, placental cells often invade the lining of the blood vessels. However, in PE, the blood flow is diminished, the arteries stay constricted, and this colonization process is only partially finished. Created in BioRender (https://www.biorender.com/) (Naidoo, P. (2025)).

In normal pregnancies, the low resistance muscular arteries are converted to a tortuous high flow low resistance system, with a 4- to 6-fold increase in arterial diameter, to meet the demands of the growing fetus [92]. However, trophoblast cell invasion is impaired in PE, and only the decidua undergo spiral artery remodeling [58,93] (Figure 6). The pathogenesis of PE can be divided into two stages: the feto-placental stage, also known as the preclinical stage, which involves defective placentation in the first and second trimesters, and the maternal stage, also known as the clinical stage, which occurs in the second and third trimesters [94]. In the later stage, poor placental perfusion causes an excessive release of inflammatory and anti-angiogenic factors into the maternal bloodstream [95]. An imbalance between the generation, release, and response to vasodilators in favor of vasoconstrictors by the endothelium results in a heightened inflammatory response [94]. Notably, there is a greater release of soluble endoglin (sEng) and the soluble version of the endothelial growth factor receptor type 1 (soluble fms-like tyrosine kinase, sFlt-1) into the maternal circulation [96]. More precisely, sEng stimulates endothelial cells in a pro-migratory and pro-angiogenic manner, whereas sFlt-1 inhibits the activation of vascular endothelial growth factor receptor type 2 (VEGFR2) in the maternal and fetal tissues [14].

## 6. Human Immunodeficiency Virus Infection

Infections such as malaria, human immunodeficiency virus (HIV), tuberculosis, and syphilis account for more than one-third of all maternal deaths worldwide [97]. HIV infection is a major contributor to poor global public health, affecting 40.1 million lives [98]. The estimated HIV prevalence rate in the South African population is approximately 14% [99]. The total number of people living with HIV (PLWH) in SA is estimated at 8.45 million in 2022. Of note, 19.6% of adults aged 15–49 years are HIV positive. More importantly, young girls in their reproductive ages of 15–24 remain at substantial risk of acquiring HIV [100]. The overall HIV prevalence in pregnancy at national level is 27.5% with the highest overall HIV prevalence occurring in the province of KwaZulu-Natal (37.1%) [101].

HIV infection is linked with a chronic inflammatory process [102]. The most advanced stage of HIV infection is acquired immunodeficiency syndrome [103]. The rate at which HIV infection progresses to AIDS depends on viral, host, and environmental factors [95,104]. Additionally, a variety of host proteins interact with HIV proteins or DNA to either limit or encourage virus replication in particular cell types. When the founder virus is transmitted, HIV replication quickly increases, followed by a notable increase in inflammatory cytokines and chemokines [105]. When the concentration of the uninfected CD4+ T-cells goes below 200 cell/mm^3^, then the infection progresses to AIDS [106].

HIV treatment involves the use of combined antiretroviral therapy (ART) to effectively suppress the viral load and to preserve/improve immune function and therefore reduces the risk of opportunistic infections and cancers [1]. Of note, ART also decreases inflammation caused by immune activation contributing to increased occurrence of cardiovascular, renal, neurological, and other end-organ diseases [1]. ART usage has reduced sexual and vertical transmission [88]. HIV infection is now a chronic, treatable condition rather than a near-death experience thanks to the invention and widespread use of powerful ARTs like highly active ART (HAART), which requires concurrent administration of at least three antiretroviral medications [88]. Globally, the risk of HIV-infected pregnant women transmitting the virus to their unborn children has drastically decreased due to greater availability to ART, however, studies proposed that the risk of PE development is heightened among treated HIV-infected women [88,107]. Despite the receipt of ART, PLWH display a high rate of non-AIDS related comorbidities. Additionally, they present with increased systemic oxidative stress due to suppression of endogenous antioxidant enzymatic mechanisms, leading to increased systemic immune activation, analogous to a PE milieu [108,109]. In a large cohort of 1038 pregnancies, while the incidence of new-onset HDP was comparable across ART classes, the risk was higher for those starting ART after 20 weeks of pregnancy than for those starting ART at conception [110]. A reduced chance of obtaining a viral load test during pregnancy is linked to starting ART during pregnancy as opposed to before [101]. Regardless of CD4 cell count, viral load, or clinical stage, the World Health Organization advises triple ART for all HIV-positive pregnant and lactating women [1,111].

There are four classes of ARTs, namely, nucleoside/nucleotide reverse-transcriptase inhibitors (NRTIs) with examples being azidothymidine, zidovudine, lamivudine, abacavir, emtricitabine, stavudine, and tenofovir; the non-nucleoside reverse-transcriptase inhibitors (NNRTIs) with examples being efavirenz, nevirapine, and etravirine; the protease inhibitors (PIs) with examples being lopinavir, ritonavir, atazanavir, indinavir, saquinavir, nelfinavir, and the integrase strand transfer inhibitors (INSTIs) with examples being raltegravir and dolutegravir [111,112]. Drug toxicities are associated with the different classes of ART and may manifest as anemia, mitochondrial toxicity, hyperlipidemia, fat redistribution, and insulin resistance [111]. Table 1 highlights some of the adverse effects of the ART classes.

## 7. The Synergy of PE and HIV Infection

Regardless of the stage of infection, HIV infection may lower fertility [115]. Considering that perinatal HIV transmission can happen in utero, during labor and delivery, or postnatally through breastfeeding, managing HIV infection during pregnancy is challenging [115]. Variable rates of unfavorable pregnancy outcomes, including increased spontaneous abortion, stillbirth, perinatal and infant mortality, intrauterine growth retardation, low birth weight, and chorioamnionitis, are linked to HIV infection [72,111,116].

HIV-infected individuals display increased systemic oxidative stress due to the suppression of endogenous antioxidant enzymatic mechanisms [108,109]. The comorbidity of HIV infection and PE remains a considerable challenge to maternal health worldwide, with the main target being low–middle income countries [117]. HIV infection causes high maternal mortality rates in communities with high HIV prevalence [118]. Although women with HIV have a far higher risk of PE than women without HIV, the risk of mother-to-child transmission among pregnant HIV-positive women has decreased dramatically globally due to increasing availability to HAART [88,107]. However, the risk of PE development is heightened among HIV-infected women receiving HAART [88].

A systemic inflammatory response and an upregulation of the immune response occurs in all pregnancies, but is significantly amplified in PE [8,119]. Excessively elevated immunological responses to innate immune system activation and other proinflammatory variables are indicative of this [119,120]. Increased proinflammatory immune cells and cytokines are the result of this immunological imbalance, together with a decline of regulatory immune cells and cytokines that eventuates in a state of inflammation [121]. As a result, inadequate invasion of trophoblasts into the myometrium and insufficient remodeling of spiral arteries leading to placental ischemia by exposing the placental site to vasoconstriction culminating in the development of PE [122]. Of note, EOPE is a proinflammatory placental state, whilst in LOPE, systemic maternal inflammation occurs [123]. Immunological deficiency, caused by HIV, may lower the incidence of PE development by reducing immune hyper-reactivity [121]. A breakdown of immune tolerance or immunological incompatibility between the mother and fetus may be associated with PE development [124]. The proinflammatory Th1 response plays a dominant role in PE; however, in combination with HIV infection and ART, the Th1 response is intensified [125]. PE risk is increased by the immunological reconstitution linked to ART usage and the toxic mechanism involving endothelial inflammation and liver damage, which leads to the loss of the protective effect [8].

Chronic immunological activation and inflammation occur in HIV infection and is prognostic of the disease development [126]. The hallmarks of chronic inflammation include immune cell metabolic dysregulation, cellular fatigue, and malfunction. Antiretroviral therapy significantly lowers immunological activation and systemic inflammation, but not to levels that are consistent with HIV naïve individuals [127]. Table 2 highlights the mechanism of action of ARTs. The use of ART leads to immune reconstitution, which increases the risk of PE development compared to treatment naïve women [88]. Notably, the HIV accessory protein Tat mimics vascular endothelial growth factors (VEGFs), preventing VEGFR-2 signaling by the VEGF-A ligand [128]. More specifically, the VEGF is unable to bind to its receptor preventing its effector function, resulting in abnormal angiogenesis. This also anticipates endothelial injury, thereby increasing the risk of PE development [128].

ART can interfere with both the severity of PE and placental development. The relationship between ART and these outcomes is complex, primarily involving various biological mechanisms that affect angiogenesis, immune response, and vascular health. Women on ART have been reported to experience higher rates of hypertension during pregnancy compared to those not receiving treatment [142,143]. Since PE is characterized by an imbalance in angiogenic factors, notably a decrease in VEGF and an increase in sFlt-1, research suggests that ART can alter this balance [125,132,139]. Specifically, protease inhibitor-based regimens have been linked to lower levels of sFlt-1 and higher levels of VEGF [144]. This shift could potentially mitigate some aspects of PE severity by promoting more favorable angiogenic conditions. Also, ART affects immune responses during pregnancy. The immunological changes induced by HIV infection can exacerbate PE through heightened inflammation. However, effective ART may help restore some degree of immune balance, which could influence the severity of PE.

Progesterone plays a crucial role in regulating placental development and angiogenesis [145]. Protease inhibitors might directly contribute to placental and uteroplacental pathology by altering their plasma. The rise in placental progesterone also plays an important role in the adaption of the maternal immune system to a predominant anti-inflammatory T-helper-2 (Th2) phenotype [138]. Some studies have found that women on protease inhibitors exhibit lower progesterone levels during pregnancy [138,146]. This decline could negatively impact placental function and contribute to adverse outcomes such as fetal growth restriction or increased risk for developing PE. Placentas from HIV-positive women receiving ART often show increased numbers of small-diameter vessels compared to those from HIV-negative controls [144,147]. While this might suggest enhanced vascularization, it is associated with poorer fetal outcomes like small for gestational age infants.

## 8. Dysregulation of Adamts13

Genetic mutation changes the protein building blocks within the ADAMTS13 enzyme. Of note, a deficiency of the VWF-cleaving protease (ADAMTS13) originates in such mutations in the ADAMTS13 gene [148]. Due to this dysregulation, an unusual dysfunctional form of the enzyme is produced. This flawed VWF processing makes it more likely that ULVWF multimers will develop, which will draw platelets and encourage the formation of microthrombi [32]. The ADAMTS13 enzyme breaks down VWF into smaller pieces, which encourages the body to create aberrant clots. Clinical signs and symptoms of TTP are caused by the multimeric form of VWF, which causes platelets to adhere to one another even when there is no damage [149]. Notably, when these clots obstruct blood channels and limit blood flow to vital organs like the brain, heart, kidneys, and placenta, they result in severe diseases.

**Figure 7:**
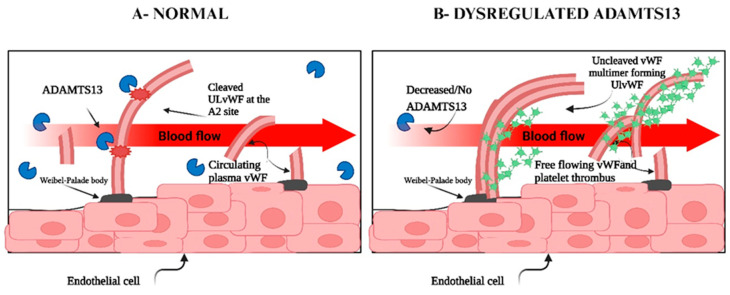
A schematic illustration depicting deficiency of ADAMTS13. (**A**) The endothelial cells’ Weibel–Palade bodies are where VWF multimers are made and stored. ADAMTS13 cleaves VWF in the platelet-rich thrombus when it is in a stretched shape due to excessive shear stress. (**B**) ULVWF bundles accumulate when VWF-dependent platelet accumulation is unchecked due to the absence of ADAMTS13 or autoantibodies that block it. (Adapted from [31] and created in BioRender (https://www.biorender.com/) (Naidoo, P. (2025))).

Two mechanisms that cause aberrant ADAMTS13 activity has been identified [150]. The most common mechanism is brought on by circulating anti-ADAMTS13 autoantibodies, which neutralize enzymatic activity and/or hasten the elimination of protease from circulation. Secondly, ADAMTS13 gene abnormalities may result in congenital TTP, where ADAMTS13 activity is less than 10% of normal levels [151]. This is also termed Upshaw–Schulman syndrome, a rare autosomal recessive disorder caused by homozygous or double-heterozygous mutation of ADAMTS13 gene [152,153]. This predisposes to the formation of unusually large forms of VWF multimers within circulation (Figure 7) culminating in intravascular platelet clumping and thrombotic microangiopathies [152]. Severe ADAMTS13 deficiency may be predisposed by specific combinations of polymorphisms or mutations; nevertheless, mutation screening has shown significant genetic variability, with the majority being limited to a single family [151]. Additionally, ULVWF multimers are generated at liver injury sites in altered vascular endothelial cells, and a reduction in ADAMTS13 activity may contribute to sinusoidal microcirculatory disruptions and the development of liver injury that ultimately results in multi-organ failure [154].

Synonymous single nucleotide variants (sSNVs) may cause protein deficiency or dysfunction pre-empting diseases [155]. Synonymous variations might cause unstable mRNA or faulty proteins by changing constitutive splice sites or activating cryptic splice sites [156]. About 200 ADAMTS13 disease-causing SNVs have been found in TTP patients; all of these were non-synonymous and found to be haplotypes [81,157]. 

A total of 376 naturally occurring sSNVs of ADAMTS13 were found in the SNP in a recent study by Jankowska et al., 2022, which included 19 from patients with USS and 357 from a healthy population [155]. Nearly 9% of the nucleotides in the ADAMTS13 mRNA are impacted by these sSNVs, with exon 25 containing the majority of them. They also revealed that many sSNVs were discovered in exons 24 to 29, which encode the Tsp1 (7–8) and CUB1–2 domains, as well as in the M domain and C-terminal portion of ADAMTS13 [155]. These SNVs alter the coding sequence, splice regulatory areas, or untranslated sequences, which results in downstream decreased plasma ADAMTS13 activity and disturbed ADAMTS13-VWF interactions [155,158]. Furthermore, multiple alternatively spliced mRNA variants have been identified, and different truncated versions of ADAMTS13 can be found in plasma [155]. Deficiency or dysfunction of ADAMTS13 due to SNVs may lead to thrombotic pathologies, including TTP, USS, myocardial infarction, and ischemic stroke [159].

## 9. Dysregulation of Von Willebrand Factor

The most prevalent of the hereditary coagulopathies is vWD, which is followed by hemophilia B, a factor IX deficiency [160]. Drug activities or adverse effects, as well as underlying systemic disease, can cause acquired coagulation abnormalities [161]. Tadu found that platelet counts were considerably lower in PE where bleeding times were significantly greater allied to D-dimer levels being significantly higher in PE compared to normotensive pregnant women [18].

**Figure 8:**
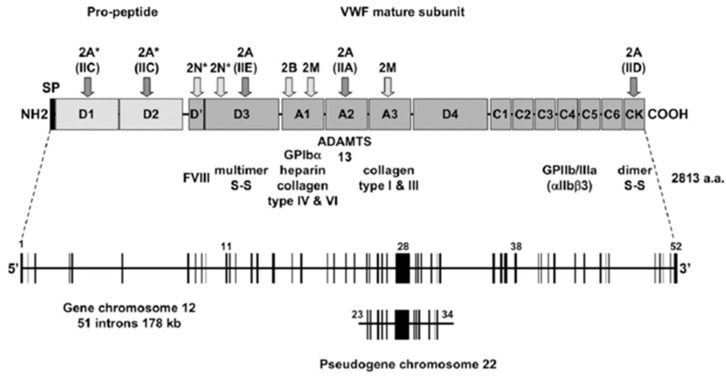
Mutations occurring at different points in VWF structure results in distinct types of vWD. The VWF precursor’s gene, pseudogene, and structure are displayed above. The signal peptide (residues 1–22), propeptide (residues 23–763), and mature subunit (residues 764–2813) make up the schematic structure of the VWF precursor (prepro-VWF). Repeats of homologous structural domains (A, C, and D) make up the pro-VWF. VWF cleavage sites for ADAMTS13, collagen, platelet GPIIb/IIIa (aIIbb3), factor VIII, and platelet glycoprotein Iba are displayed. The locations of the mutations causing VWD type 2 are shown by arrows (adapted from [164]).

Mutations in VWF disrupt multimer assembly, secretion, and/or catabolism thereby influencing bleeding [162]. Mutations in VWF result in deficiencies in the VWF protein predisposing mild to severe bleeding, a disorder known as Von Willebrand disease (vWD) (Figure 8) [163]. This is an autosomal inherited mucocutaneous bleeding disorder [64]. Defects in the secretion or intravascular clearance of VWF multimers results in dysregulation of vWD type 1 [64,162], whilst defects in the assembly or intravascular proteolysis of VWF multimers influence vWD type 2A or 2B [162]. Missense mutations affecting platelet binding or FVIII-binding are responsible for the four sub-types, 2A, 2B, 2M, and 2N, whilst mutations resulting in a lack of VWF expression predominate in recessive type 3 vWD [64]. Increased VWF levels predispose atherothrombotic complications [163]. Apart from its function in hemostasis, VWF may induce downstream cell-signaling pathways associated with angiogenesis, inflammation, cell apoptosis, and metastasis as well as vascular wall thickening [163].

## 10. Adamts13 in HIV Infection

In HIV-associated TTP, the dysfunction of vascular endothelial cells due to HIV infection can lead to local thrombin generation and consumption of ADAMTS13 [165]. Notably, HIV-associated TTP occurs in the setting of profound CD4 deficiency with altered ADAMTS13 protease activity complicated by myocardial infarction and stroke [166,167]. ADAMTS13 response may be impaired in HIV infection. In a study by Graham, HIV viral load correlated with both ADAMTS13 antigen and activity [167]. More specifically, men with acute HIV infection had significantly higher levels of ADAMTS13 activity compared to HIV naïve controls [167]. Additionally, both acute and chronic untreated HIV infection exhibited higher ADAMTS13 activity compared to chronic treated infection [167]. ADAMTS13 levels are lower in HIV-infected compared to HIV naïve individuals [153]. Autoantibodies formed against ADAMTS13 are present in PLWH because of an impaired immune system [153]. HIV infection itself can lead to an increase in VWF levels and a decrease in ADAMTS13 activity, which may contribute to a prothrombotic state [33].

## 11. Von Willebrand Factor in HIV Infection

A significant increase in VWF levels is linked to the advancement of HIV illness; there is a positive association between VWF levels and plasma viral load, and higher levels of VWF antigen are associated with a higher risk of mortality [167]. Despite an apparent equivalent rise in the quantity and activity of its regulatory protease (ADAMTS13), VWF antigen quantity and adhesive activity are raised after HIV infection [167]. HIV-1 infection causes a procoagulant condition because the immune and coagulation systems are closely related and stimulate one another [168]. Untreated HIV infection is associated with increased endothelial activation, inflammation, and coagulation [169]. VWF release, sustained attachment to the vascular wall, and self-assembly into strings and fibers facilitate platelet adherence and endothelial cell activation [167]. There is a positive association between VWF levels and plasma viral load, and a significant increase in VWF levels is linked to the advancement of HIV illness [167]. Following successful ART, levels of the VWF antigen and other endothelial activation indicators also decline [167]. Furthermore, in PLWH with African ancestry, the increased VWF content might be a contributing factor to endothelial cell damage [170]. 

## 12. Von Willebrand Factor and HIV Treatment

Inflammation and coagulation system activation are linked to HIV infection and continue throughout antiretroviral therapy [167]. VWF is released when endothelial activity brought on by HIV infection occurs. It either enters the bloodstream or adheres to vessel walls, where it self-assembles into strings and fibers to facilitate platelet adherence [171]. The study by Graham and Chen [167] noted that plasma viral load positively correlated with VWF adhesive activity, whilst elevated levels of circulating VWF occurs in treated HIV infected individuals, however this study included a male population [167]. Of note they had also demonstrated that VWF antigen levels decrease post ART [167]. Notably, patients receiving abacavir did not have increased levels of plasma coagulation markers such as VWF compared to patients who did not receive an abacavir containing regimen [172]. Combination ART includes regimens that contain protease inhibitors that reduces but do not normalize levels of VWF [172]. Of note, over time the effect of VWF on the intact endothelium with allied platelet adhesion promotes atherosclerosis and an increased cardiovascular risk in PLWH [173]. 

## 13. Adamts13 in Pregnancy

ADAMTS13 concentration is dependent on age, being lowest in neonates and in individuals above 65 years of age in physiological conditions [35]. Pregnancy is characterized by deep placentation; physiological placentation is characterized by the invasion of the uterine spiral arteries by extravillous trophoblast cells arising from anchoring villi [174]. Notably, the differentiation and invasive activity of the trophoblast cells is tightly controlled spatially and temporally also regulated by multiple factors to enable proper placentation, thus defects in trophoblast invasion are associated with severe pregnancy-related complications [175]. 

Human placentae’s trophoblast cells and villous endothelium cells express full-length proteolytically active ADAMTS13 which reaches their peak levels during the first three months of pregnancy, then decrease during the second and third trimesters [176]. ADAMTS13 mRNA and protein are expressed in human normal placenta and decidua throughout the pregnancy, as well on trophoblast and fetal blood vessel endothelium [176]. For a cell to migrate, proteolysis of the extracellular matrix is mediated by the matrix metalloproteases [177,178]. A decreased plasma ADAMTS13 activity occurs at 12 to 16 weeks gestation and by the third trimester it decreases progressively to 23% with an increased sensitivity to thrombotic microangiopathies [TMAs; the presence of hemolytic anemia (destruction of red blood cells), low platelets, and organ damage due to blood clots in capillaries and small arteries] occurring within the second trimester of pregnancy [35,179]. TMAs result in abnormalities in the blood vessel walls of arterioles and capillaries with resultant microvascular thrombosis. Hemolytic uremic syndrome and TTP are primary forms of TMAs [153]. Thrombotic thrombocytopenic purpura is caused by severe deficiency of ADAMTS13 due to acquired autoantibodies or genetic mutations [166]. The most common form of HUS (typical HUS) follows a diarrheal illness caused by *Shiga* toxin-producing *Escherichia coli*, whereas atypical HUS is associated with abnormal host susceptibility to complement-mediated damage. Moreover, TTP is three times more common in females, and half of those affected are pregnant or postpartum [180]. ADAMTS13 may be either pro- or anti-angiogenic depending on its local microenvironment [181].

Throughout gestation, proper regulation of VWF by ADAMTS13 is critical for maintaining normal blood flow in the placental vasculature. Imbalances in this regulatory mechanism may lead to excessive platelet adhesion and aggregation within the placental circulation, resulting in thrombotic complications such as placental infarction or abruption [182]. These conditions can impair fetal growth and development by compromising the exchange of nutrients and oxygen between the mother and fetus [93]. Dysregulation of this enzyme can lead to thrombotic complications and contribute to pregnancy-related disorders that affect placental function and fetal well-being [32]. 

## 14. Adamts13 in Preeclampsia

**Figure 9:**
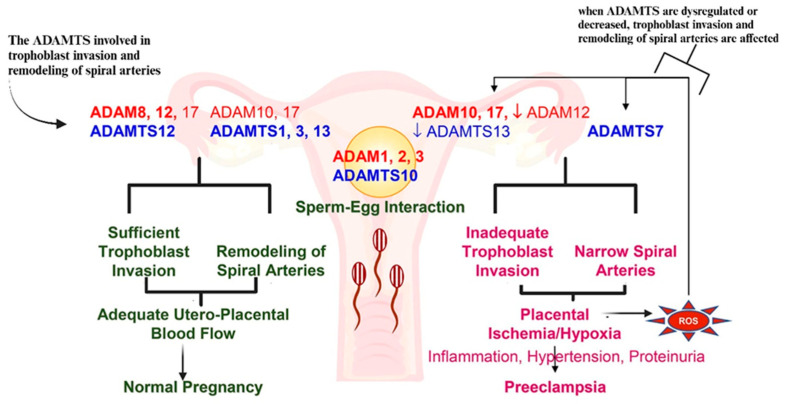
Illustration of the expression of different ADAMs and ADAMTS in hypertensive pregnancy and ADAMTS13’s role in the development of preeclampsia (adapted from [14]).

Placental villi express significantly lower levels of ADAMTS13 protein in PE compared to normotensive pregnancies [176]. A reduction in ADAMTS13 synthesis and secretion within the placentae of women with severe PE may be associated with hypertension-related placental ischemia and tissue hypoxia [176]. Functional changes in ADAMTS13 proteases induce maternal and fetal complications by stimulating extracellular matrix development [183]. Aref and Goda [184] reported that plasma ADAMTS13 activity was significantly reduced in PE compared to normal pregnant women and non-pregnant women [178]. Partial deficiencies of ADAMTS13 have been observed in diseases sharing inflammatory states, including cardiovascular diseases, severe sepsis, and septic shock [185,186]. There is significant association between ADAMTS13 activity levels and EOPE, whereas severe PE was associated with increased levels of P-selectin [35]. ADAMTS13 has begun to be identified as a prognostic and/or diagnostic marker of other diseases, such as those related to inflammatory processes, liver damage, metastasis of malignancies, sepsis, and different disorders related to angiogenesis [21] (Figure 9), and hence may be a useful predictor marker for severe PE development.

Placental ADAMTS13 is produced at its maximum levels during the first trimester of pregnancy, when placentation takes place, and decreases throughout the second and third trimesters of gestation [176]. Throughout the pregnancy, ADAMTS13 mRNA are expressed in normal placentae, deciduae, trophoblast, and fetal blood vessel endothelium [176]. In severe PE, the hypoxic microenvironment causes a significant decrease in ADAMTS13 levels in placental villous tissues [149,176]. The decrease in ADAMTS13 production following placental explant exposure to hypoxic circumstances lends credence to this theory [176]. ADAMTS13 stimulates trophoblast cell migration, invasion, proliferation, and network formation during pregnancy [176], indicating a function for the ADAMTS13 protease in both healthy pregnancy and PE’s impaired placentation.

ADAMTS13 has recently been discovered to be a prognostic and/or diagnostic marker of various other illnesses, including those associated with inflammation, liver damage, cancer metastases, sepsis, and various angiogenesis-related conditions [21], and hence may be a useful predictor marker for PE development since it reflects an antiangiogenic milieu. Preeclampsia is associated with an early decline of ADAMTS13 activity independently of VWF concentration, thus resulting in an increase in circulating VWF with high placental microthrombotic risk [12,35]. Notably, ADAMTS13 activity that is lower than 10% leads to microvascular thrombosis, accounting for the placental dysfunction and the possible underlying pathophysiology of PE [187].

## 15. Von Willebrand Factor in Pregnancy

Hormonal influences across gestation contributes to an increase in VWF shifting hemostasis to a procoagulant state to compensate for the anticipated hemorrhage during parturition [188]. Von Willebrand disease is caused by either a quantitative or qualitative defect in VWF secretion [189]. During pregnancy, significant changes occur in the hemostatic system of several plasma proteins, especially at term gestation [190]. The levels of VWF antigen increases across the trimesters of pregnancy [191]. Although VWF levels rise and peak during the third trimester, women with vWD are at risk of early pregnancy bleeding, as well as postpartum hemorrhage [188]. The VWF and FVIII attain high levels during normal pregnancies, while in VWD patients the pattern is variable [192].

## 16. Von Willebrand Factor Levels in Preeclampsia

The VWF antigen levels are significantly higher in PE compared to normal pregnant and non-pregnant women [184]. The source of increased VWF levels in PE is likely to be the endothelium [191]. Significantly higher levels of VWF antigen and activity from the endothelium were also noted in PE compared to normotensive pregnancy [191]. The presence of increased amounts of active VWF in PE emanates from the decreased levels of ADAMTS13 activity. This reduction in ADAMTS13 activity causes biologically active ULVWF multimers to circulate in patients with PE [184]. In a study conducted by Deng and Bremme [193], the levels of VWF were higher in PE than in normal pregnancy within the second and third trimesters. Of note, patients with severe PE had elevated levels of VWF five weeks postpartum [193]. 

HELLP syndrome, or hemolysis, high liver enzymes, and low platelet count syndrome, is a serious pregnancy complication that frequently co-occurs with PE [194,195]. Maternal mortality due to HELLP is reported to be between 1 and 30% [195]. Hemolytic anemia, TTP, and organ damage due to micro-clots are characteristics of the wide range of TMAs that include the HELLP syndrome [196]. Notably, pregnant women without a history of hypertension or proteinuria may develop HELLP syndrome [196]. HELLP syndrome affects placentation in the early stages of pregnancy and is linked to involvement of the coagulation cascade and liver [194]. Intravascular hemolysis (H), increased liver enzymes (EL), and decreased platelet count (LP)—including lactate dehydrogenase > 600 U/L, aspartate aminotransferase ≥ 70 U/L, and thrombocytopenia < 100.0 G/L—are the hallmarks of the full form of HELLP syndrome [196]. The majority of cases occur between weeks 27 and 37 of pregnancy, while 30% of cases occur after delivery. The most common symptoms include nausea, vomiting, and right upper abdominal quadrant or epigastric discomfort [195].

HELLP syndrome impairs placentation during the preliminary stages of pregnancy and is associated with the involvement of hepatic and coagulation cascades [194]. Maternal vascular endothelium activation brought on by placental ischemia increases the production of anti-angiogenic factors that enter the bloodstream [197]. Endothelial damage is common as a result of these anti-angiogenic agents [198]. Vasospasm, platelet aggregation, and increased endothelial damage result from platelet adhesion to injured endothelium, which initiates the coagulation cascade [195]. Damage of vascular endothelial cells emanate from anti-angiogenic factors together with elevated levels of active VWF [194]. Women with severe HELLP and multi-organ failure have high blood quantities of thrombin–inhibitor complexes, which exacerbate coagulation activation [194]. A coexistence of HELLP and placental abruption has been reported [194]. In patients with the HELLP syndrome, systemic endothelial damage, complement dysregulation, and elevated serum levels of active multimeric VWF leads to TMAs and multi-organ microvascular injury [195].

## 17. Adamts13 in the Duality of PE and HIV Infection

Decreased ADAMTS13 levels occur in the pathological states of diabetes, TTP, and PE [199]. Notably, a deficiency of ADAMTS13 occurs in other inflammatory conditions such as cardiovascular diseases [200], severe sepsis and septic shock [179], myocardial infarction, severe *Plasmodium falciparum* malaria [201], alcoholic hepatitis, and anti-phospholipid syndrome [35]. P-selectin (P-sel) and Tsp1 are involved in the regulation of ADAMTS13 protease activity towards VWF production by colocalizing with VWF in WPBs and platelet α-granules. [35]. P-selectin levels are increased in women with PE [202]. Inflammatory cytokines such as interleukin-4, interleukin-6, interleukin-1β, interferon-γ, and tumor necrosis factor-α may inhibit ADAMTS13 proteolytic activity and/or its expression in HSCs and endothelial cells [35].

The study conducted by Funderburg, investigated ADAMTS13 levels in HIV-infected women and found that they were significantly lower in PLWH compared to healthy controls [172]. Bashir reports similar findings [153]. In contrast, Graham observed that ADAMTS13 antigen and activity were significantly higher in PLWH than uninfected people; however, pregnant women were excluded from this population [167]. Autoantibodies to ADAMTS13 are additionally present in PLWH because of an impaired immune system [153]. HIV infection itself can lead to an increase in VWF levels and a decrease in ADAMTS13 activity, which may contribute to a prothrombotic state [33].

When compared to normotensive pregnant women, PE is associated with an early decline of ADAMTS13 activity independently of VWF concentration, thus resulting in an increase in circulating VWF with high placental microthrombotic risk [12,35]. Notably, ADAMTS13 activity lower than 10% could lead to microvascular thrombosis, which could explain placental dysfunction and the possible underlying pathophysiology of PE [187]. Women with obstetric anti-phospholipid syndrome can develop placental diseases, such as PE, a diagnosis associated with reduced ADAMTS13 levels [12]. Additionally, Venou and Varelas [203] reported that women with PE had decreased ADAMTS13 activity [197]. The mRNA expression of ADAMTS13 in the placenta with its protein abundance and proteolytic activity are higher at the first trimester of pregnancy but are lower at term in normal pregnancies [14]. In PE, ADAMTS13 expression in the placental villous tissue is reduced, possibly emanating from placental ischemia, oxidative stress, and endotheliosis [14], implicating a role of ADAMTS13 protease in the pathogenesis of pregnancy-associated vascular remodeling [14]. 

The protein level of the proteoglycanases viz., ADAMTS1, ADAMTS4, ADAMTS12, and ADAMTS13, are also reported to be lower in maternal and umbilical cord blood but all except ADAMS13 are higher in the placental tissue of preeclamptic women compared to normal pregnancies [14]. The maternal systemic signs arise from soluble factors which are released from the placenta in response to oxidative stress [12]. The relationship between ARTs and ADAMTS13 levels is sparse, however, understanding this interaction is important for managing the overall health of women receiving ART.

## 18. Von Willebrand Factor in the Duality of PE and HIV Infection

In contrast to normal pregnancy, women with PE exhibit heightened platelet activation including consumptive thrombocytopenia, increased mean platelet volume, and generation of platelet microparticles [204]. PE platelets are dysfunctional, hyper-activated, and prothrombotic although they become less able to aggregate [204]. Therefore, it is likely that platelet activation contributes to the prothrombotic state of PE [204]. It is plausible that the increased maternal inflammatory response in PE predisposes to extensive endothelial cell activation which is known to elevate soluble thrombomodulin, E-selectin, and VWF levels [184]. 

HIV-1 proteins are associated with endothelial dysfunction and vascular remodeling [205]. Notably, VWF is considered to be a marker of endothelial dysfunction [206]. Raised VWF levels corresponds with greater platelet adhesiveness thereby promoting a thrombotic tendency especially in stenotic vessels, leading to increased shear stress [207]. Evidence suggests that HIV infection promotes chronic arterial inflammation and injury that promotes endothelial dysfunction, atherosclerosis, and thrombosis in the HIV treatment naïve population [208]. VWF levels may correlate with the degree of endothelial dysfunction in this chronic inflammatory state, which would increase thrombosis even further. Platelets and HIV infection are linked, as platelets are capable of internalizing HIV particles, leading to platelet-bound HIV-1 infected permissive cells [207]. Numerous factors could contribute to the rise in platelet reactivity, including endothelial dysfunction and chronic inflammatory conditions [209,210]. The diverse metabolic effects of the various ART drug classes, such as the direct harm that ART causes to endothelial cells, could potentially be the cause of this rise [207]. Notably, VWF levels are also correlated with overall immunological state, viral load, and CD4 count; increasing VWF levels are associated with lower CD4 count and higher viral load [207]. Patients with sepsis, DIC, liver disorders, plasmodium falciparum infection, transplantation, immunosuppression with cyclosporin, and sickle cell disease have been found to have ULVWF multimers with or without significantly decreased levels of ADAMTS13 [211].

The amount of VWF that can be activated by Ristocetin is a measure of the functionality of VWF and can be determined using the RCo assay (VWF:RCo) [212]. Notably, plasma VWF levels and the VWF:RCo are significantly higher in PE compared to healthy pregnant and non-pregnant women [184]. In contrast, Hulstein and Heimel [212] found that VWF:RCo was not significantly increased in PE compared to a healthy pregnant group. ADAMTS13 activity is also significantly lower in severe PE compared to mild PE, whilst VWF antigen levels and VWF:RCo are significantly elevated in severe PE compared to mild PE [184]. Molvarec and Rigó Jr [213] concluded that the plasma ADAMTS13 activity is normal in PE despite the increased VWF:Ag levels [207]. In contrast, the study by Stepanian and Cohen-Moatti [35] had shown that individuals with the lowest levels of ADAMTS13 had a significantly increased risk of PE development, independent of VWF:Ag levels [34]. 

Other types of TMAs have been connected to complement activation and inflammation, which results in endothelial dysfunction and an overabundance of ULVWF multimers [214]. It is plausible to assume that since PE is also associated with a complement dysregulation [215,216], enhanced inflammation [119] and endothelial dysfunction [108], ADAMTS13, and VWF levels may also be dysregulated. Of note, in patients receiving abacavir, NNRTIs have elevated VWF levels whilst combination ART regimens containing PIs reduce but do not normalize levels of VWF [172]. Additionally, short-term treatment with ART reduces markers of endothelial dysfunction such as VWF, with no differences between PIs and NNRTIs [217].

## 19. Coupled Adamts13 and Von Willebrand Factor in HIV-Associated Preeclampsia

**Figure 10:**
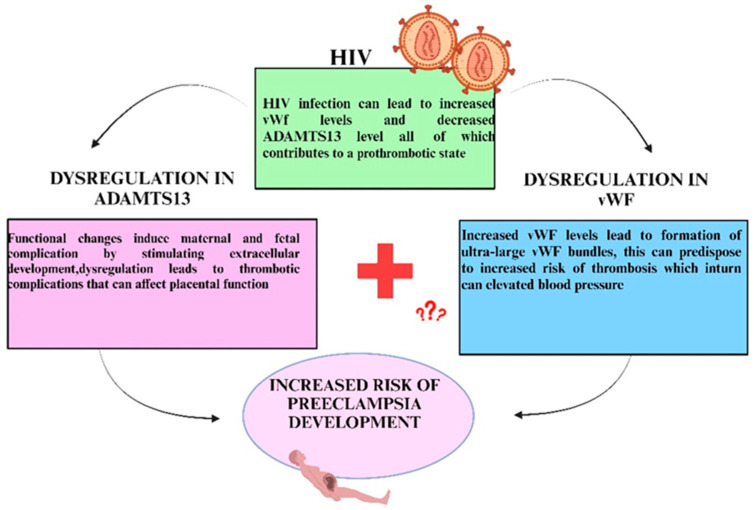
The possible relationship between HIV infection, ADAMTS13, and VWF. Created in BioRender (https://www.biorender.com/) (Naidoo, P. (2025)).

Significant uteroplacental and vascular remodeling by proteolytic enzymes and metalloproteinase is necessary for pregnancy-related processes [14]. Of note, HIV infection causes an imbalance of ADAMTS13/VWF homeostasis. In women where there is a decrease in the ADAMTS13 enzyme due to either a mutation in the gene or autoantibodies formed against ADAMTS13, VWF is unable to break up into smaller molecules, thus resulting in endothelium-anchored ULVWF chains precipating uncontrolled thrombosis, increasing blood pressure and the risk of PE development [32]. Figure 10 illustrates the possible relationship between ADAMTS13 and VWF in PLWH and the development of PE.

During hypoxia, endothelial cells are activated, promoting the release of WPBs which facilitate blood coagulation by initiating thrombus formation [218]. P-selectin is stored in WPBs and is increased during hypoxia [219]. Vascular injury and inflammation induce WPBs to simultaneously release VWF [220] and P sel translocation [221]. P-selectin dysregulation occurs in the pathogenesis of HIV-associated coagulopathy [222]. Also, P-selectin levels are attenuated in PE compared to normotensive pregnancies [223]. Therefore, it is plausible that VWF and P-selectin release is altered in the hypoxic state of PE where endothelial cells injury and heightened inflammation predominates. Moreover, P-selectin is an adhesion receptor, hence it may alter placentation.

**Figure 11:**
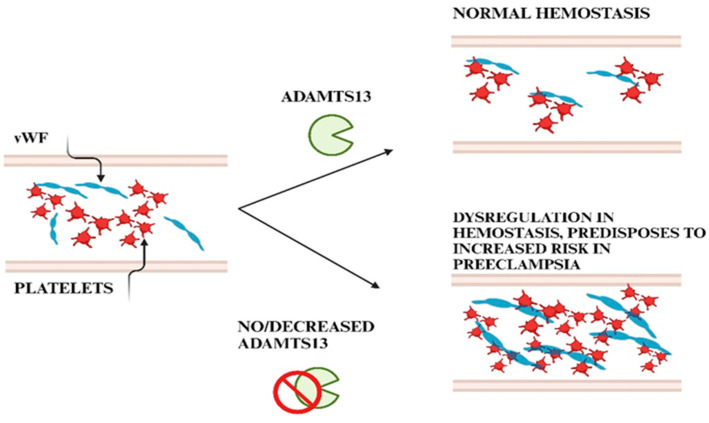
Schematic diagram illustrating possible relationship between ADAMTS13 and VWF molecules. VWF—Von Willebrand Factor, ADAMTS13—a disintegrin and metalloprotease with thrombospondin type motif 13. Created in BioRender (https://www.biorender.com/) (Naidoo, P. (2025)).

The deposition of VWF occurs in venous and arterial subendothelial matrices implicating their role in thrombogenicity [224]. Of note, microthrombi may also occur in capillaries. The fine balance between VWF and ADAMTS13 ensures that circulating VWF is hemostatically active but not prothrombotic [225]. It is however disrupted in PE where VWF binds to the GP Ib-IX-V-complex to activate platelets predisposing to a pro-coagulative activity [63,226]. Of note, in PE with associated hypercoagulability, placental-derived extracellular vesicles of platelet and endothelial cells are significantly elevated [227] (Figure 11).

Additionally, studies have found that PE is associated with early decreased levels of ADAMTS13, independently of VWF, contributing to the increase in circulating VWF in PE and possibly enhancing the placental microthrombotic risk [12]. The association of dysregulated ADAMTS13 and ART in HIV infection may predispose to the risk of PE development; whereby the ULVWF multimers increase clotting which in turn upregulate blood pressure, hence it is necessary to investigate these effects in the synergy of HIV infection and PE. Table 3 highlights a few studies that examined the levels of ADAMTS13 and VWF in HIV or PE, however, no study seemed to examine the effect of both analytes in conjunction with the duality of both diseased states. The combined effect of a decrease in ADAMTS13 and being HIV positive would have fatal effects, further increasing maternal mortality and morbidity. Identification of pregnant women who have HIV as well as a dysregulation in the ADAMTS13 protease could potentially affect pregnancy outcomes, thus research into these proteins will enable better management of this comorbidity.

## 20. COVID-19 in the Synergy of HIV and Preeclampsia in Relation to Adamts13 and Von Willebrand Factor

**Figure 12:**
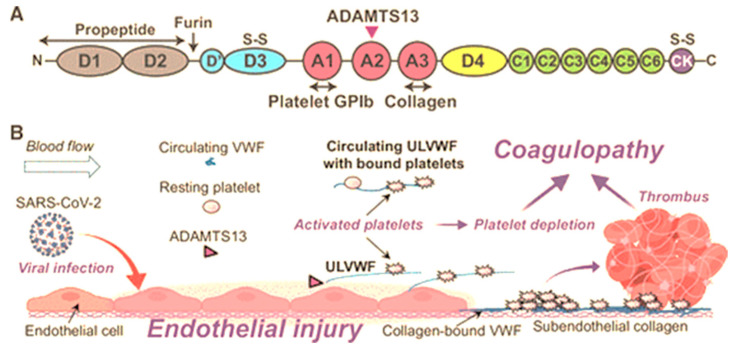
Schematic illustration of viral infection, endothelial injury, the VWF/ADAMTS13 axis, and COVID-19 coagulopathy. (**A**) Illustration of VWF domain arrangement, with A-domains’ key functions. (**B**) Coagulopathy because of the combined effects of viral infection with endothelial injury (adapted [235]).

COVID-19 is caused by the highly contagious novel coronavirus SARS-CoV-2 [233]. The SARS-CoV-2 infection directly causes endothelial damage and dysregulation of the immune response [234]. A high incidence of thrombotic events has been observed in severe cases of COVID-19 [235]. SARS-CoV-2 infection was reported to induce hypertension and preeclampsia-like symptoms in pregnant women [236]. Intrauterine infection caused by COVID-19 can alter ACE2 expression, promoting a preeclamptic state [237]. The SARS-CoV-2 infection promotes circulating proinflammatory cytokines with induction of endothelial secretion of ULVWF that causes an imbalance in VWF/ADAMTS13. Zhang and Bignotti [238] noted significantly elevated plasma levels of VWF in COVID-19 patients compared to healthy controls concomitant with lower plasma ADAMTS13 activity in patients with critical COVID-19 [238]. The insufficiency of ADAMTS13 to cleave ULVWF may result in hypercoagulability, including spontaneous thrombus formation in blood vessels and VWF adhesion onto subendothelial collagen exposed during endothelial injury (Figure 12) [235]. Increased endothelial cell activation and Weibel–Palade body exocytosis in severe COVID-19 lead to markedly increased plasma VWF levels [68]. Patients with severe COVID-19 have been found to have significantly higher plasma VWF antigen (VWF:Ag) and activity (VWF:RCo and VWF:CB) levels, which is consistent with the idea that severe COVID-19 is linked to notable endothelial cell activation [117]. In severe cases of infection, SARS-CoV-2 mimics angiotensin II-mediated PE through the utilization of ACE2 to cause endothelial dysfunction and hypertension [108,117]. A possible risk factor for SARS-CoV-2 infection and subsequent PE development is the upregulation of ACE2 in pregnancy [117]. 

## 21. Conclusions

This review highlights the conceptual framework underlying the hemostatic balance involving ADAMTS13 and VWF dysregulation in PLWH and PE. ADAMTS13 downregulation predisposes PE development, independent of VWF:Ag levels. An ADAMTS13 activity lower than 10% could lead to microvascular thrombosis, possibly explaining placental dysfunction in PE. Moreover, P-sel and Tsp1 participate in the regulation of ADAMTS13 protease activity required for the formation of VWF. Additionally, inflammatory cytokines inhibit ADAMTS13 proteolytic activity/expression in endothelial cells. The increased maternal inflammatory response in PE predisposes extensive endothelial cell activation thereby upregulating VWF levels. The plasma concentration of VWF in PE is significantly increased compared to normotensive pregnancies reducing ADAMTS13 expression within the ischemic placental microenvironment, suggesting a role of ADAMTS13 protease in the pathogenesis of pregnancy-associated vascular remodeling in PE. ART may be involved in increasing platelet reactivity, such as the direct damage of endothelial cells by ART. It was noted that HIV infection promotes the induction of endothelial secretion of ULVWF that causes an imbalance in VWF/ADAMTS13; this raised VWF levels coincides with greater platelet adhesiveness, promoting a thrombotic tendency, which may predispose maternal mortality.

The involvement of ADAMTS13 and VWF and/or HIV infection may predispose an increased risk of PE development, hence it is necessary to investigate these effects in the synergy of HIV infection and PE. Women with HIV infection require ART and if left untreated, the results would be fatal to both mother and fetus. The combined effect of a decrease in ADAMTS13 and increased VWF while being HIV positive would have fatal effects, further increasing maternal mortality and morbidity. Identification of pregnant women who are HIV-infected and have a dysregulation in the ADAMTS13 enzyme could potentially have an effect on pregnancy outcomes.

## 22. Future Directions

We recommend conducting a large-scale study investigating the allelic and genotypic differences in single nucleotide polymorphisms of ADAMTS13 and VWF in pregnant women of African ancestry. Furthermore, future investigations should include correlations between ADAMTS13 and VWF together with their downstream signaling effects. Additionally, further research is warranted to explore the interplay between HIV infection, ART, and the pathogenesis of PE as it may guide the development of safer ART regimes during pregnancy.

Further research involving single nucleotide polymorphisms of VWF and ADAMTS13 should be conducted on a large sample cohort in women of different ethnicities and ancestry.

## Figures and Tables

**Table 1:** Adverse effects of art classes.

ARTClass	Effects	Reference
Nucleoside/Nucleotide Reverse Transcriptase Inhibitors (NRTIs)	Zidovudine and stavudine have been shown to induce mitochondrial toxicity and oxidative stress in platelets, leukopenia, elevation of liver enzyme levels, elevation of lactic acid level Abacavir—hypersensitivity reactions such as fever, rash, myalgia, arthralgia, malaise	[27,113,114]
Non-Nucleoside Reverse Transcriptase Inhibitors (NNRTIs)	Efavirenz and nevirapine—induce hepatic enzyme induction, alter platelet metabolism, central nervous system toxicity, and psychosis and rash	[27,113,114]
Protease Inhibitors (PIs)	Impairing platelet function Induce endothelial dysfunction Alter the balance of pro- and anti-thrombotic factors Gastrointestinal upset, rash Indinavir—nephrolithiasis, hypertension	[27,113,114]
Integrase Strand Transfer Inhibitors (INSTIs)	Raltegravir and dolutegravir is associated with changes in lipid metabolism, endothelial function, gastrointestinal upset, and hepatitis	[27,114]

**Table 2:** Mechanism of action of different arts on preeclampsia development.

ART Class	Mechanism of Action	Reference
Non-nucleoside reverse transcriptase inhibitor (NNRTIs)	They bind in a non-competitive way to HIV-1 reverse transcriptase enzyme and inhibit the conversion of viral RNA into DNA. Restores immune response and are elevated during oxidative stress. Dysregulate NF-κB transcription factors and hence decrease MMP-9 and VEGF expression. Dysregulate the immunoexpression of angiopoietin, endoglin, and PlGF. Decrease tight junction proteins such as claudin-1, occludin, zonula occluden-1, and junctional adhesion molecule-1 which increase vascular permeability.	[117,129,130,131,132]
Nucleoside/nucleotide reverse transcriptase inhibitors (NRTIs)	These directly block HIV-1 reverse transcriptase enzyme from converting viral RNA into DNA. They reconstitute immune response. Decrease endothelial cell proliferation, migration via defective tyrosine kinase receptor, and VEGFR-2 signaling. Exacerbate mitochondrial oxidative stress, and this increase in ROS generation pre-empts trophoblast apoptosis and thus predisposing PE and/or IUGR development.	[117,133,134,135]
Protease inhibitors (PIs)	Inhibit HIV-1 protease, inhibiting the transformation of immature HIV particles to mature HIV particles. They restore immune response. They deplete uNK cells. However, they lower progesterone in trophoblast cells, hence impeding invasion following decreased expression of the transcription factor STAT3. This leads to a dysregulated uterine decidualization, incomplete trophoblast cell invasion, and defective spiral artery remodeling. They also decrease VEGF, PlGF, angiopoietin-2, interferon-gamma, and MMP-9 in decidual cells. They decrease endothelial cell proliferation and migration and cause defective tyrosine kinase receptors and VEGFR-2 signaling. Moreover, PIs also elevate mitochondrial oxidative stress which leads to increased ROS generation elevating trophoblast apoptosis and predisposing PE and/or IUGR development.	[117,136,137,138,139]
Integrase strand transfer inhibitors (INSTIs)	Prevents HIV replication by blocking integrase which is used to insert viral DNA into the host CD4 cell.	[140]
HAART	Based on immune reconstitution. HAART dysregulates NF-κB transcription factors, hence decreasing MMP and VEGF expression. HAART also is implicated in an increase in sFlt-1 and sEng with concomitant decrease in PlGF and VCAM-1 expression.	[125,141]

**Table 3:** Studies that examined ADAMTS13 and/or VWF in HIV and/or PE.

Article	ADAMTS13	VWF	PE	HIV	HIV-Associated Preeclampsia	Reference
Increased plasma von Willebrand factor antigen levels but normal von Willebrand factor cleaving protease (ADAMTS13) activity in preeclampsia	NORMAL	↑	√	-	-	[207]
Von Willebrand factor antigen and ADAMTS13 activity assay in pregnant women and severe preeclamptic patients	NORMAL	↑	√	-	-	[228]
ADAMTS13, FVIII, von Willebrand factor, ABO blood group assessment in preeclampsia	↓	↑	√	-	-	[229]
Von Willebrand Factor and ADAMTS13: A Candidate Couple for Preeclampsia Pathophysiology	↓	↑	√	-	-	[35]
Increased VWF antigen levels and decreased ADAMTS13 activity in preeclampsia	↓	↑	√	-	--	[184]
High Levels of von Willebrand Factor and Low Levels of its Cleaving Protease, ADAMTS13, are Associated with Stroke in Young HIV-Infected Patients	↓	↑	-	√	-	[207]
von Willebrand Factor is elevated in HIV patients with a history of thrombosis	-	↑	-	√	-	[230]
Expression of ADAMTS13 in Normal and Abnormal Placentae and Its Potential Role in Angiogenesis and Placenta Development	↓	-	√	-	-	[176]
ADAMTS 1, 4, 12, and 13 levels in maternal blood, cord blood, and placenta in preeclampsia	↓	-	√	-	-	[231]
Von Willebrand Factor Adhesive Activity and ADAMTS13 Protease Activity in HIV-1-Infected Men	NORMAL	↑		√	-	[167]
Elevated Plasma von Willebrand Factor Levels Are Associated With Subsequent Ischemic Stroke in Persons with Treated HIV Infection	NORMAL	↑		√	-	[232]
Alterations in the von Willebrand factor/ADAMTS-13 axis in preeclampsia	↓	↑	√	-	-	[20]

↑ increased concentration/activity. ↓ decreased concentration/activity. √—the disease state examined.

## Data Availability

No new data were created.
